# Factors Associated with Nutritional Risk in Colorectal Cancer Patients Undergoing Chemotherapy: A Secondary Analysis of a Cross-Sectional Study

**DOI:** 10.3390/nursrep16010027

**Published:** 2026-01-16

**Authors:** Yan Xu, Qianqian Du, Ningxiang Luo, Shurong Lai, Zhijun Zhou, Meifen Zhang

**Affiliations:** 1 Department of Gastrointestinal Surgery, The First Affiliated Hospital of Sun Yat-sen University, Guangzhou 510000, China; xuyan63@mail.sysu.edu.cn (Y.X.); laishr@mail.sysu.edu.cn (S.L.); 2 School of Nursing, Sun Yat-sen University, No. 74, Zhongshan Road II, Guangzhou 510000, China; duqianq@mail2.sysu.edu.cn; 3 Department of Surgical Nursing Teaching and Research, The First Affiliated Hospital of Sun Yat-sen University, Guangzhou 510000, China; luonx@mail.sysu.edu.cn; 4 Department of Medicine, University of Oklahoma Health Campus, 975 NE 10th Street, BRC 1213, Oklahoma City, OK 73104, USA

**Keywords:** nutritional risk, dietary pattern, food intake, nutrition knowledge, nursing

## Abstract

**Background/Objectives**: Our previous study showed that the dietary structure is imbalanced in a majority of colorectal cancer patients receiving chemotherapy. These patients had higher risk of developing malnutrition. In the present study, we aimed to identify factors associated with nutritional risk in this cohort of patients. **Methods**: We performed a secondary analysis of a dataset that was originally collected to identify the factors that are associated with an imbalanced dietary structure in patients receiving chemotherapy for colorectal cancer. Nutritional risk was evaluated by using an NRS-2002 form. Binary logistic regression was used for multivariate analysis. **Results**: Among the 178 CRC patients enrolled in this study, 60 (33.7%) had nutritional risk. Patients with nutritional risk exhibited lower intake of grains, potatoes, vegetables, fruits, beans, nuts, and oils compared to those without risk. Multivariate analysis showed that non-surgery (95% CI: 0.130–0.914, *p* = 0.032) and high dietary structure score (95% CI: 0.808–0.944, *p* = 0.001) are associated with lower nutritional risk in CRC patients receiving chemotherapy. **Conclusions**: CRC patients receiving chemotherapy have moderate risk of developing malnutrition. Dietary structure score and surgery are associated with malnutrition in CRC patients receiving chemotherapy. Education on proper dietary structure is a potential strategy to mitigate nutritional risk in CRC patients undergoing chemotherapy. These findings highlight the need for personalized nutritional support to optimize patient outcomes.

## 1. Introduction

Colorectal cancer (CRC) is the third most prevalent cancer type and the second leading cause of cancer mortality globally [1]. Surgery, chemotherapy, and targeted therapy are currently the most important treatment options for CRC. Chemotherapy is the backbone treatment for CRC patients after surgery. Except for a small number of early-stage CRC patients, who may not need adjuvant chemotherapy post-surgery, around 80% of CRC patients would need chemotherapy at some point throughout the treatment process [2]. Studies showed that among CRC patients receiving chemotherapy, 60–70% had nutritional risk [3], indicating these patients may develop malnutrition, body weight loss, muscle wasting, and cachexia in the near future, all of which would impair quality of life and lead to poor treatment outcomes. In addition to indicating the risk of malnutrition, nutritional risk assessed by NRS-2002 reflects a broader concept that incorporates disease severity and metabolic stress. Meanwhile, nutritional risk is associated with an inflammatory status and increased energy expenditure [4,5], leading to worse prognosis in cancer patients [6]. A study showed that CRC patients with nutritional risk had a higher incidence of side effects during chemotherapy, including gastrointestinal symptoms such as nausea and vomiting. In addition, chemotherapy drugs can easily cause patients to feel fatigue and low, and their emotional and physiological discomfort can seriously affect their quality of life. The preoperative nutrition status can predict the short- and long-term treatment outcomes of surgery in CRC patients [7]. Therefore, identifying factors associated with nutritional risk may enable the implementation of proactive strategies to prevent its development in these patients.

Several factors have been identified to be related to the development of nutritional risk in CRC patients undergoing chemotherapy, including advanced tumor stages and chemotherapy-related adverse effects, such as fatigue, mucositis, and loss of appetite [8]. Meanwhile, cancer-induced muscle wasting or sarcopenia can lead to nutritional risk and poorer outcomes [9]. Elevated level of C-Reactive Protein (CRP), a biomarker of systemic inflammation, is related to impaired nutrient utilization and increased catabolism, leading to malnutrition and sarcopenia in CRC patients [10]. The Glasgow Prognostic Score, which combines CRP and albumin level, can predict nutritional risk and overall prognosis of cancer patients [11]. Intriguingly, evidence shows that hypoalbuminemia is independent of systemic inflammation in reflecting nutritional risk [12]. Hypoalbuminemia has been recognized as a sign of disease-related malnutrition, often aggravated by inflammation and poor diet [12]. While CRC patients have high nutritional risk during the perioperative period, the association between the alteration of dietary structure and nutritional risk in these patients remains elusive.

Some patients are temporally not allowed to eat because of the surgery or cancer-induced intestinal obstruction. For these patients, parenteral nutrition support is necessary to provide the nutrients needed for daily life. Most patients can eat freely after the recovery from surgery. However, due to the changes in the structure and function of the digestive system after surgery, patients need a certain amount of time to establish new diet patterns. Meanwhile, most chemotherapy has obvious side effects, which inevitably influence the eating habit or dietary structure of these patients. Additionally, some patients are concerned about cancer and the adaptation of the new digestive system after surgery, so they are selective about the food they eat.

Therefore, most patients undergoing chemotherapy are not eating a balanced diet because of loss of appetite, taste alteration, oral discomfort, and diarrhea [13], causing an imbalanced diet. These patients are likely to develop malnutrition, which in turn prolongs the patient’s recovery time. Some patients may have heard about the importance of nutritional support, but they may not believe in it or may be unwilling to act. These individuals are also more likely to have an imbalanced diet and are at increased risk of developing malnutrition [14].

The current study presents a secondary analysis of an existing dataset that was originally generated to examine determinants of dietary imbalance in colorectal cancer patients undergoing chemotherapy [15]. The original study evaluated dietary structure, but did not investigate nutritional risk. The current study addressed a different hypothesis by evaluating the nutritional risk of these patients. To identify factors associated with developing malnutrition in these patients, we performed binary logistic regression analysis. Early detection of patients at risk of malnutrition and guidance on their dietary structure may reduce the risk of malnutrition, thus improving the treatment efficacy and prognosis.

## 2. Materials and Methods

### 2.1. Participants

This is a secondary analysis of an existing dataset established by our group [15]. All participants received chemotherapy for the treatment of CRC, and volunteered to participate in this study. Informed consent was obtained from all patients. The inclusion criteria for participating in this study are as follows: CRC confirmed by pathological diagnosis; age ≥ 18 years old; currently receiving chemotherapy; no mental illness, clear consciousness, and ability to answer questions; having an informed understanding of this study, and agreement to participate in this study. The exclusion criteria for this study are as follows: severe surgical treatment complications; severe bone marrow suppression; major organ dysfunction, combined with other malignant tumors; previous esophagectomy; total gastrectomy; total colectomy and other serious digestive and absorptive function-restricted diseases; and those who are unable to take food by mouth and whose main way of taking food is tube feeding. Ethical approval was obtained from the Ethics Committee of FAH-SYSU in the original study [15].

### 2.2. Demographic and Clinical Characteristics of Patients

Patients’ demographic data were collected, including age, gender, job, insurance type, height, weight, and BMI value. Meanwhile, the clinical characteristics of patients were also collected, including surgery, chemotherapy cycles, chemotherapy regimen, tumor stage, and surgery.

### 2.3. Evaluation of Nutritional Risk

We assessed patients’ nutritional risk using the NRS-2002 scale. NRS-2002 is the first nutritional risk screening tool established through evidence-based studies [16]. NRS-2002 is a reliable tool for nutritional risk screening and was recommended by the European Society for Clinical Nutrition and Metabolism [17,18]. The NRS-2002 nutritional score includes three scoring items: (1) disease severity: score range 0–3 points; (2) nutritional status impairment: score range 0–3 points; and (3) age, score range 0–1 point. Total score = disease severity score + nutritional status impairment score + age score. The total score ranges from 0 to 7 points. A total score ≥ 3 indicated malnutrition or nutritional risk, warranting nutritional intervention or close monitoring, whereas a score of 0–2 indicated no nutritional risk at the time of assessment.

### 2.4. Evaluation of Dietary Structure

Evaluating dietary structure involves two steps: A dietary survey and a scoring assessment.

#### 2.4.1. Dietary Survey

Patients’ food intake over the past month during chemotherapy was assessed using the Guangdong Provincial Food Frequency Questionnaire and the Retrospective Dietary Survey-Assisted Reference Food Atlas [19,20].

#### 2.4.2. Dietary Structure Assessment

The dietary structure was evaluated using the Chinese Food Pagoda (CHFP) scoring method, which is based on dietary guidelines for Chinese residents. This method groups 10 food categories and assigns a score based on how closely a patient’s intake matches the recommended levels. Each category has a maximum of 5 points, with a total possible score of 45. A higher score indicates a better dietary structure.

(1) Common food groups include cereals and potatoes, vegetables, fruits, aquatic products, dairy, and soybeans.

- If intake meets or exceeds the recommended level, full points are given.
- If intake is below the recommended level, points are given proportionally.

(2) Special food groups include poultry, eggs, oil, and salt.

- If intake exceeds the maximum recommended amount, 0 points are given.
- If intake is within or below the recommended range, full or proportional points are given using the following formula: Score = Full score × [1 − (Actual intake − 0-point standard) ÷ (0-point standard − Full-point standard)].

### 2.5. Nutrition Knowledge–Attitude–Practice Evaluation

This study utilized the Nutrition Knowledge–Attitude–Practice (KAP) Questionnaire for patients with gastrointestinal tumors, developed by Jing Zhang [21]. The questionnaire includes the following components:

1. Nutrition Knowledge Dimension: Comprising 17 Items, with a Total Possible Score of 17. Correct Answers Are Scored as 1 Point, While Incorrect or Uncertain Answers Are Scored as 0
2. Nutrition Attitude Dimension: Consisting of 5 Items, Scored Using a 5-Point Likert Scale with a Total Score of 20. “Strongly Disagree” Is Scored as 0 Points and “Strongly Agree” as 4 Points
3. Nutrition Practice Dimension: Includes 8 Items, with a Total Score of 32 Points
  (1) Items 1, 2, 3, 7, and 8 are positively worded and scored on a 5-point Likert scale, where “Never” equals 0 points and “Always” equals 4 points.
  (2) Items 4, 5, and 6 are negatively worded and scored in reverse, where “Never” equals 4 points and “Always” equals 0 points.

The total score range of the questionnaire is 0 to 69. The Cronbach’s α coefficient of the questionnaire is 0.822, and the test–retest reliability is 0.79.

### 2.6. Sample Size Calculation

According to previous studies, the incidence of nutritional risk in patients undergoing chemotherapy for digestive tract tumors was about 30–60% [22]. Stata software (version 15.0) was used to calculate the two-sided test level at 0.05, and the allowable error was 10%. When the nutritional risk incidence rate is set as 45%, and considering a 30% loss rate, the minimum required sample size is 138 cases.

### 2.7. Statistical Analyses

Statistical analyses of this study were performed using SPSS (IBM SPSS Statistics, version 25.0). Descriptive variables were shown as the mean ± standard deviation (SD), and number percentages (%). Bivariate correlation analysis was performed using the Pearson correlation coefficient for normally distributed data and Spearman rank-order correlation for the non-normal data. The Chi-squared test or Student’s *t*-test was applied for the univariate analysis. Binary logistic regression was used for multivariate analysis. The statistical significance level was *p* < 0.05.

## 3. Results

### 3.1. Clinicopathological Characteristics and Demographic Information of the Patients Enrolled in the Study

**Figure 1:**
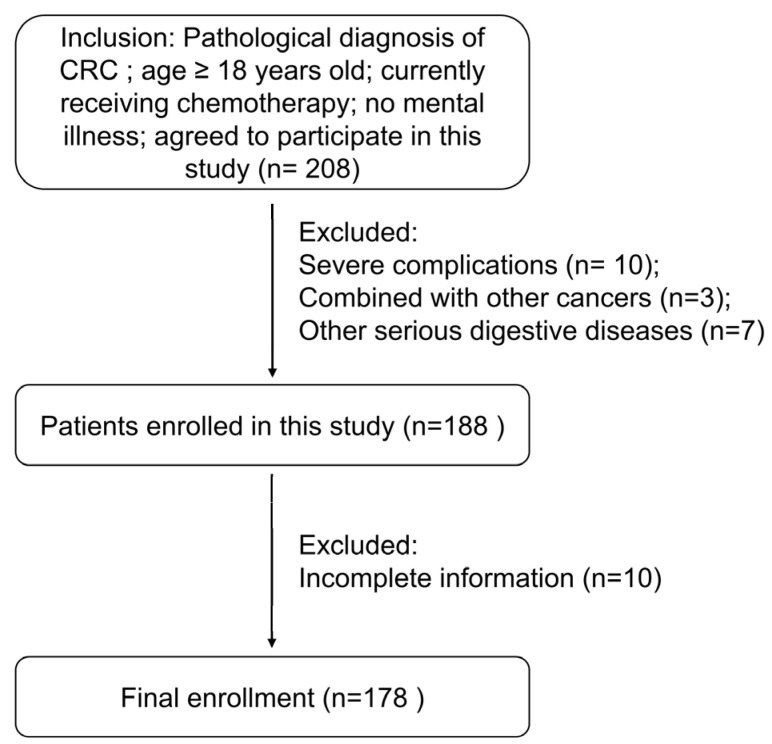
A flow diagram illustrating patient selection criteria.

This is a secondary analysis of a cross-sectional study. Patient selection criteria are shown in Figure 1. The clinicopathological characteristics and demographic information of the patients enrolled in this study have been reported in our previous publication [15]. For readability, detailed information is provided in Supplemental Tables S1 and S2.

### 3.2. Nutritional Risk of Patients Receiving Chemotherapy for CRC

Among the 178 patients, 60 patients had nutritional risk, accounting for 33.7%. The overall average nutritional risk score of patients was (2.05 ± 1.25) points (Table 1).

### 3.3. Univariate Analysis of Potential Factors of Nutritional Risk in Patients Receiving Chemotherapy for CRC

We analyzed the association between nutritional risk and social characteristics, including sex, age, BMI, residence, marriage status, academic degree, employment, income, payment method, and commercial insurance status. Only BMI is associated with nutritional risk in CRC (Table 2).

We further analyzed the association between nutritional risk and clinicopathologic characteristics, including stage, lymphatic metastasis, distant metastasis, surgery, chemotherapy regimen and chemotherapy cycles. We found that these clinicopathologic characteristics are not significantly related to nutritional risk (Table 3). Meanwhile, we evaluated the nutrition knowledge, awareness, and action in these patients and found that patients with nutritional risk had lower nutrition knowledge (Table 4). These results indicate that BMI and nutrition knowledge are associated with nutritional risk in CRC.

**Figure 2:**
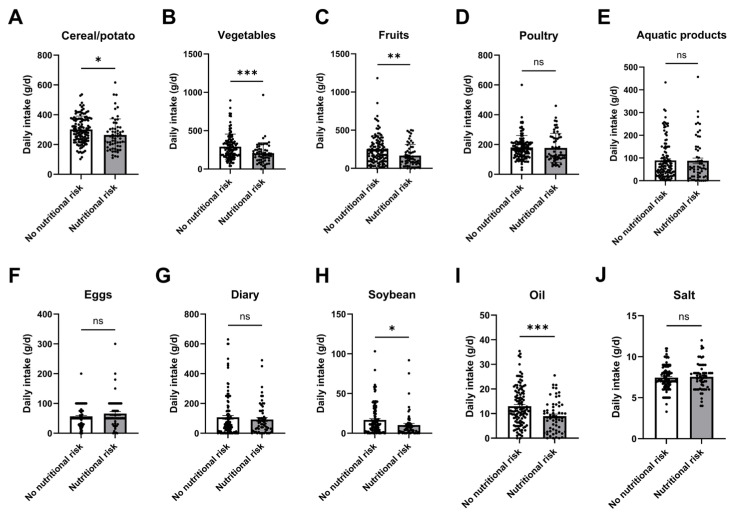
Comparison of dietary structure of CRC chemotherapy patients. Daily average uptake of (**A**) cereal/potato, (**B**) vegetables, (**C**) fruits, (**D**) poultry, (**E**) aquatic products, (**F**) eggs, (**G**) dairy, (**H**) soybean, (**I**) oil, and (**J**) salt in CRC chemotherapy patients with or without nutritional risk. *, *p* < 0.05; **, *p* < 0.01; ***, *p* < 0.001; ns, not significant.

The *t*-test was used to analyze the relationship between nutritional risk and dietary structure in patients undergoing CRC chemotherapy. The dietary structure score of CRC chemotherapy patients in the nutritional risk group was lower than that in the non-nutritional risk group (*t* = 3.851, *p* < 0.001). The dietary intake of cereals, potatoes, vegetables, fruits, soybeans, and oils was significantly lower in patients of the nutritional risk group compared to that of the non-nutritional risk group (*p* < 0.05), as shown in Figure 2 and Table 5.

### 3.4. Multivariate Analysis of Potential Factors of Nutritional Risk in Patients Receiving Chemotherapy for CRC

We further performed multivariate analysis to evaluate factors of nutritional risk in patients receiving chemotherapy for CRC. Age and BMI were excluded from multivariate analysis because age and BMI are well-known factors associated with nutritional risk, which have been included into the NRS2002 scoring system. Although univariate analysis indicated that surgery, chemotherapy regimen, number of chemotherapy cycles, and cancer stage were not significantly associated with nutritional risk in this cohort of CRC patients undergoing chemotherapy, these factors are clinically relevant and may act as co-factors [23,24]. Therefore, we included these potential covariates, along with dietary structure score and nutrition knowledge score, in the multivariate analysis. The results showed that surgery and dietary structure score are significantly associated with nutritional risk. Those that have undergone surgery and those that had a low dietary structure score have higher nutritional risk (Table 6).

## 4. Discussion

Most CRC patients need chemotherapy, which can cause appetite loss, intestinal flora imbalance, and taste bud changes, thereby exacerbating malnutrition and leading to nutritional risk, a higher incidence of side effects and reduced tolerance to treatment [25]. Our study showed that 33.7% of CRC chemotherapy patients had nutritional risk. This is consistent with studies by Xavier Hébuterne et al. [26] and Gheorghe et al. [27], which reported a nutritional risk rate of 39.3% and 26.8% in CRC patients, respectively. To identify factors that are associated with nutritional risk, we conducted a secondary analysis of the dataset initially collected to evaluate dietary structure in CRC patients receiving chemotherapy.

We found that surgery is associated with the development of nutritional risk in CRC patients with chemotherapy. Although our univariate analysis did not reveal significant association between surgery and nutritional risk, it has been demonstrated that some clinically relevant variables should also be included in multivariate models, even if they do not reach statistical significance in univariate analysis [28]. Therefore, we included surgery for multivariate analysis and revealed that surgery is indeed associated with the development of nutritional risk in CRC patients. Surgery for colorectal cancer often involves partial resection of the bowel, which can lead to reduced absorptive surface area, altered gut motility, and changes in the gut microbiota. In addition, perioperative complications, such as anastomotic leakage, infections, or prolonged ileus, can further impair oral intake and nutrient absorption. Gut microbiota has been shown to play critical roles in influencing the risk of anastomotic leakage for colorectal cancer surgery [29]. Furthermore, pain, fatigue, and appetite loss post-surgery may contribute to insufficient caloric and protein intake. Therefore, perioperative malnutrition in colorectal cancer is a multifactorial and dynamic process influenced by surgical stress, systemic inflammation, reduced oral intake, and metabolic alterations. Major colorectal surgeries, such as colectomy and total mesorectal excision, can induce acute protein catabolism and transient nutritional deterioration, particularly in the early postoperative period. Prior studies have shown that perioperative nutritional support and Enhanced Recovery After Surgery (ERAS) programs improve postoperative outcomes, highlighting the importance of nutritional assessment around surgery [30,31]. Moreover, preoperative sarcopenia and postoperative nutritional decline are associated with reduced chemotherapy tolerance. Patients with advanced disease who do not undergo surgery may also experience malnutrition due to tumor burden and inflammation, emphasizing the need for contextual interpretation of surgical history.

Dietary structure is another important factor. We found that poor dietary structure is associated with nutritional risk in these patients. The Dietary Guidelines for Chinese Residents (2022) recommend a consumption of more than 25 types of food per week and more than 12 types of food per day for a reasonable dietary mix. Making dietary choices based on the dietary pagoda helps reduce random food selection, ensuring a more balanced intake of energy, thermogenic nutrients, and trace elements. It promotes scientific meal planning, optimizing nutrient composition and minimizing the risks associated with imbalanced diets. Proper nutrition not only requires appropriate dietary proportions to meet the body’s daily energy and nutrient needs, but also considers dietary principles and cooking methods to enhance food intake and overall meal experience for patients. Studies have shown that greater dietary diversity is linked to better dietary structure and a reduced risk of all-cause mortality [32]. We observed that many CRC patients exhibited distinct food habits and preferences. The change in dietary pattern in these patients is probably due to a combination of physiological, psychological, and treatment-related factors. These patients usually focus on several certain types of food while ignoring the balance of other food components. We found that the dietary intake of cereals, potatoes, vegetables, fruits, soybeans and oils was significantly lower in patients of the nutritional risk group. A study reported that high milk intake (>100 mL/day) is associated with an increased risk of hand–foot syndrome (OR = 2.711, 95%CI: 1.195–6.816), while high egg consumption (>100 g/day) is associated with bone marrow suppression in CRC chemotherapy patients [33]. These associations may be mediated by alterations in the plasma metabolome, particularly lipid profiles. However, it remains elusive whether the findings can be applied to nutritional risk. In our study, we did not observe any significant differences in milk or egg consumption between participants with and without nutritional risk. We found that although the energy supply ratio of carbohydrates and fats in CRC patients undergoing chemotherapy aligns with recommended guidelines, the actual intake of these macronutrients is insufficient to meet daily energy requirements. As fundamental thermogenic nutrients, inadequate consumption of carbohydrates and fats can exacerbate fatigue and lethargy, hindering energy storage. The insufficient intake of fiber and vitamins makes patients feel fragile and ultimately contributes to nutritional risk. Improving the dietary structure of CRC chemotherapy patients not only increases the intake of essential nutrients but also enhances their quality of life. Studies have shown that dietary adjustments can effectively reduce nutritional risks and contribute to better overall well-being [22,34]. Early nutritional assessment and intervention (i.e., dietary counseling, oral supplements, or enteral nutrition) can improve treatment tolerance, reduce side effects, and enhance survival [35]. A study showed that educational and nutrition interventions in gastric cancer patients receiving chemotherapy significantly increased the compliance rate of chemotherapy and improved the overall physical status of these patients [36]. Therefore, education on dietary structure represents a potential strategy to prevent nutritional risk in CRC patients treated with chemotherapy.

Next, we asked whether a specific chemotherapy regimen or treatment cycle is associated with increased nutritional risk, and found that there is no significant difference in terms of nutritional risk between different regimens or cycles of chemotherapy. A possible explanation is that the adverse nutritional impact of chemotherapy may be more related to general treatment exposure and individual patient susceptibility, rather than the specific regimen or number of cycles. In other words, the metabolic stress, appetite changes, and gastrointestinal side effects induced by chemotherapy appear to occur across regimens, leading to a broadly similar risk of malnutrition regardless of protocol.

Furthermore, while some studies found that advanced stages are associated with nutritional risk [37], our study did not find a significant correlation between tumor stages and nutritional risk. From a clinical perspective, nutritional risk in patients with colorectal cancer is not solely determined by tumor stage, but is strongly influenced by treatment-related factors and baseline nutritional reserves. Systemic therapies commonly used across disease stages, including chemotherapy and targeted agents, frequently cause anorexia, nausea, diarrhea, and mucositis, leading to reduced oral intake and increased metabolic demands irrespective of tumor burden. Moreover, interindividual variability in baseline nutritional status, body composition, comorbidities, and inflammatory responses can substantially modify nutritional vulnerability. As a result, patients with both early- and advanced-stage disease may experience comparable degrees of appetite loss, metabolic alterations, and treatment-related toxicities, thereby attenuating stage-dependent differences in nutritional risk. In addition, standardized supportive care and nutritional interventions implemented during chemotherapy, such as antiemetic prophylaxis, symptom management, and dietary counseling, may further mitigate expected stage-related disparities.

This study also has some limitations. This study focused exclusively on a Chinese population and evaluated dietary patterns based on the Chinese Dietary Guidelines (CHFP), without comparison to other dietary patterns. Consequently, the generalizability of the findings to colorectal cancer populations in diverse cultural contexts with different staple diets remains uncertain. Future studies should compare multiple dietary patterns to determine which dietary structures are most effective in mitigating chemotherapy-related adverse effects and improving treatment outcomes within specific cultural contexts. Given that this is a secondary analysis, potentially valuable variables were absent from the original dataset, such as chemotherapy side effect severity and physical status of the patients. Additionally, while nutritional intervention is important for those undergoing gastrointestinal surgery [38,39], the specific types of nutritional intervention for these patients warrant further study [40,41,42]. Furthermore, although the minimum required sample size was calculated to ensure adequate statistical power, we ultimately included all eligible cases available during the study period. Including more cases than the minimum requirement increases the precision of the estimates and reduces sampling error, thereby strengthening the robustness of the findings, but it may also waste resources and induce unnecessary stress in the participants.

## 5. Conclusions

In summary, nutritional risk is prevalent among CRC patients undergoing chemotherapy and is closely linked to dietary patterns. Addressing nutritional risk in this population is essential for improving treatment efficacy, quality of life, and overall survival. Surgery and low dietary structure score are significantly associated with nutritional risk in CRC patients undergoing chemotherapy. This study highlights the necessity of nutritional risk screening in CRC patients undergoing chemotherapy. Incorporating dietary counseling and nutritional support may help reduce the prevalence of nutritional risk and ultimately improve patient outcomes.

## Figures and Tables

**Table 1:** Detailed total and dimensional nutritional risk scores of the patients.

Item	Min	Max	Score ($\bar{x}$ ± s)
Nutritional risk score	0	7	2.05 ± 1.25
Disease severity score	0	3	1.1 ± 0.32
Nutritional status impairment score	0	3	0.89 ± 1.15
Age score	0	1	0.09 ± 0.31

**Table 2:** Univariate analysis of social characteristics in patients with colorectal cancer that received chemotherapy.

Factor	No nutritional Risk (*N* = 118)	Nutritional Risk (*N* = 60)	*X* ^2^	*p*
Sex			0.129	0.846
Male	76 (64.4)	37 (61.7)		
Female	42 (35.6)	23 (38.3)		
Age (years)			3.179	0.204
18–39	10 (8.5)	10 (16.7)		
40–59	67 (56.8)	28 (46.7)		
60–77	41 (34.7)	22 (36.7)		
BMI			8.352	<0.001
<18.5	0 (0.0)	19 (31.7)		
18.5–23.9	71 (60.2)	33 (55.0)		
≥24	47 (39.8)	8 (13.3)		
Residence			6.939	0.074
City	73 (61.8)	27 (45.0)		
County	14 (11.9)	10 (16.7)		
Town	17 (14.4)	8 (13.3)		
Country	14 (11.9)	15 (25.0)		
Marriage status			7.367	0.410
Married	110 (93.2)	53 (88.3)		
Single, divorced, or widowed	8 (6.8)	7 (11.7)		
Academic degree			2.551	0.636
Bachelor or above	13 (11.0)	8 (13.3)		
Community College	18 (15.3)	10 (16.7)		
High school	32 (27.1)	15 (25.0)		
Middle school	36 (30.5)	13 (21.7)		
Primary school	19 (16.1)	14 (23.3)		
Employment			0.595	0.440
Yes	11 (9.3)	4 (6.7)		
No	107 (90.7)	56 (93.3)		
Income (RMB/month/person)			1.661	0.436
<3500	49 (41.5)	19 (31.7)		
3500–8000	53 (44.9)	32 (53.3)		
>8000	16 (13.6)	9 (15.0)		
Payment method			4.352	0.226
Public care	16 (13.5)	7 (11.7)		
Employee	41 (34.7)	15 (25.0)		
Rural Cooperative	42 (35.6)	31 (51.6)		
Urban insurance	19 (16.2)	7 (11.7)		
Commercial insurance			0.093	0.903
Yes	30 (25.4)	14 (23.3)		
No	88 (74.6)	46 (76.7)		

**Table 3:** Univariate analysis of clinicopathologic characteristics in patients with colorectal cancer that received chemotherapy.

Factor	No Nutritional Risk (*N* = 118)	Nutritional Risk (*N* = 60)	*X* ^2^	*p*
Stage			1.941	0.379
I–II	20 (17.0)	13 (21.7)		
III	54 (45.8)	21 (35.0)		
IV	44 (37.2)	26 (43.3)		
Lymphatic metastasis			0.163	0.686
Yes	74 (62.7)	35 (58.3)		
No	44 (37.3)	25 (41.7)		
Distant metastasis			0.382	0.536
Yes	44 (37.3)	26 (43.3)		
No	74 (62.7)	34 (56.7)		
Post-surgery			3.053	0.121
Yes	89 (75.4)	52 (86.7)		
No	29 (24.6)	8 (13.3)		
Regimen			1.037	0.856
CapeOX	89 (75.4)	43 (71.7)		
FOLFIRI	8 (6.8)	5 (8.3)		
Others	21 (17.8)	12 (20.0)		
Chemotherapy cycle			2.247	0.468
<5	74 (62.7)	32 (53.3)		
5–10	28 (23.7)	17 (28.4)		
>10	16 (13.6)	11 (18.3)		

**Table 4:** Univariate analysis of knowledge–awareness–action score on nutrition in patients with colorectal cancer that received chemotherapy.

Factor	No Nutritional Risk (*N* = 118)	Nutritional Risk (*N* = 60)	*t*	*p*
Nutrition knowledge	10.8 ± 2.9	9.9 ± 3.1	2.022	0.045
Nutrition awareness	16.3 ± 2.5	15.5 ± 2.7	1.891	0.060
Nutrition action	21.5 ± 3.3	20.9 ± 3.7	1.072	0.285

**Table 5:** Analysis of dietary structure of CRC chemotherapy patients.

Diet Composition	Recommended Intake (g/d)	Actual Intake (g/d)		*t*	*p*
		No Nutritional Risk (*N* = 118) $\bar{x}$ ± *s*	Nutritional Risk (*N* = 60) $\bar{x}$ ± *s*		
Cereal/potato	>325	300.76 ± 90.28	264.63 ± 107.91	2.360	0.019
Vegetables	>400	291.18 ± 164.20	205.46 ± 138.26	3.664	<0.001
Fruits	>275	243.29 ± 186.53	166.58 ± 145.11	2.784	0.006
Poultry	<100	180.54 ± 79.46	177.10 ± 97.01	0.237	0.813
Aquatic products	>50	89.62 ± 83.79	87.54 ± 95.22	0.149	0.882
Eggs	<50	56.89 ± 31.10	66.15 ± 50.44	−1.303	0.196
Dairy	>400	106.70 ± 136.99	92.58 ± 106.83	0.698	0.486
Soybean	>30	16.87 ± 18.66	10.33 ± 16.73	2.368	0.019
Oil	<30	12.99 ± 7.99	8.97 ± 6.44	3.386	0.001
Salt	<5	7.44 ± 1.43	7.56 ± 1.73	−0.482	0.630

**Table 6:** Multivariate analysis of nutritional risk in patients with colorectal cancer that received chemotherapy.

Factors	*OR*	*95% CI*	*p*
Surgery			0.032
Yes	Reference		
No	0.345	0.130–0.914	0.032
Chemotherapy regimen			0.965
CAPEOX	Reference		
FOLFIRI	1.155	0.294–4.539	0.836
Others	0.947	0.373–2.407	0.909
Chemotherapy cycles			0.504
<5	Reference		
5–10	1.521	0.650–3.562	0.334
>10	1.865	0.578–6.022	0.297
Stage			0.596
I	Reference		
II	0.915	0.125–6.675	0.930
III	0.640	0.105–3.889	0.628
IV	1.161	0.197–6.853	0.869
Dietary structure score	0.873	0.808–0.944	0.001
Nutrition knowledge score	0.922	0.818–1.038	0.180

## Data Availability

The original contributions presented in this study are included in the article/Supplementary Materials. Further inquiries can be directed to the corresponding authors.

## Supplementary Materials

The following supporting information can be downloaded at https://www.mdpi.com/article/10.3390/nursrep16010027/s1: Table S1. Clinicopathological characteristics and treatment information; Table S2. Demographic information of the patients enrolled in the study.

## Public Involvement Statement

There was no public involvement in any aspect of this research.

## Guidelines and Standards Statement

This manuscript was prepared in accordance with the STROBE (Strengthening the Reporting of Observational Studies in Epidemiology) guidelines for cross-sectional studies.

## Abbreviations

CRC	Colorectal cancer
BMI	Body mass index
NRS-2002	Nutrition Risk Screening 2002
CHFP	Chinese Food Pagoda
CRP	C-Reactive Protein
