# Supplementary material for: Factors Associated with Nutritional Risk in Colorectal Cancer Patients Undergoing Chemotherapy: A Secondary Analysis of a Cross-Sectional Study
Source: Nurs Rep. 2026 Jan 16;16(1):27. doi: 10.3390/nursrep16010027 (PMC12845464; doi:10.3390/nursrep16010027)
Supplement: Supplementary file 1 [file nursrep-16-00027-s001.zip › nursrep-4027009-supplementary.pdf]

Supplemental Table S1. Clinicopathological characteristics and treatment information

Factor	*N*	Percentage（%）
Stage		
I	5	2.8
II	28	15.7
III	75	42.1
IV	70	39.4
Lymphatic metastasis		
Yes	133	74.7
No	45	25.3
Distant metastasis		
Yes	70	39.3
No	108	60.7
Regimen		
CapeOX	132	74.2
FOLFIRI	13	7.3
Others	33	18.5
Chemotherapy cycle		
< 5	91	51.1
5-10	60	33.7
> 10	27	15.2
Post-Surgery		
Yes	141	79.2
No	37	20.8

Supplemental Table S2. Demographic information of the patients enrolled in the study

Factors	*N*	Percentage (%)	Factors	*N*	Percentage / ($x \pm s$)
Sex			Age（years）		54.6 ± 11.5
Male	113	63.5	18-39	20	11.2
Female	65	36.5	40-59	95	53.4
			60-77	63	35.4
BMI			Residence		
<18.5	19	10.7	City	100	56.2
18.5-23.9	104	58.4	County	24	13.5
24-27.9	46	25.8	Town	25	14.0
≥28	9	5.1	Country	29	16.3
Marriage status			Income（RMB/month）		
Married	163	91.6	<3500	25	14.0
Single	5	2.8	3500-8000	85	47.8
Divorced or widowed	10	5.6	>8000	68	38.2
Academic degree			Insurance method		
Bachelor or above	21	11.8			
Community College	28	15.7	Public care	23	12.9
High school	47	26.4	Employee	56	31.5
Middle school	49	27.5	Rural Cooperative	73	41.0
Primary school	33	18.5	Urban insurance	26	14.6
Commercial insurance					
Yes	44	24.7			
No	134	75.3			
